# The occurrence of antibiotic resistance genes in the microbiota of yak, beef and dairy cattle characterized by a metagenomic approach

**DOI:** 10.1038/s41429-021-00425-2

**Published:** 2021-06-09

**Authors:** Weiwei Wang, Xiaojuan Wei, Lingyu Wu, Xiaofei Shang, Fusheng Cheng, Bing Li, Xuzheng Zhou, Jiyu Zhang

**Affiliations:** 1 Key Laboratory of New Animal Drug Project of Gansu Province, Lanzhou, Gansu Province 730050 PR China; 2 Key Laboratory of Veterinary Pharmaceutical Development, Ministry of Agriculture, Lanzhou, Gansu Province 730050 PR China; 3 Lanzhou Institute of Husbandry and Pharmaceutical Sciences, Chinese Academy of Agricultural Sciences, Lanzhou, Gansu Province 730050 PR China

**Keywords:** Antimicrobial resistance, Computational biology and bioinformatics

## Abstract

Drug resistance has been partly driven by the overuse of antimicrobials in agricultural animal feed. Better understanding of antibiotic resistance in bovine gut is needed to assess its potential effects based on metagenomic approach and analysis. In this study, we collected 40 fecal samples to explore drug resistance derived from antibiotic use in the bacterial community by an analysis of the diversities and differences of antibiotic-resistant genes (ARGs) in the gut microbiota from yak, beef, and dairy cattle. Overall, 1688 genes were annotated, including 734 ARG subtypes. The ARGs were related to tetracyclines, quinolones, β-lactam, and aminoglycosides, in accordance with the antibiotics widely used in the clinic for humans or animals. The emergence, prevalence, and differences in resistance genes in the intestines of yaks, beef, and dairy cattle may be caused by the selective pressure of different feeding patterns, where yaks were raised without antibiotics for growth promotion. In addition, the abundance of ARGs in yak was lower than in beef and dairy cattle, whereas the abundance of integron, a kind of mobile genetic elements (MGEs) was higher in yaks than those in beef and dairy cattle. Furthermore, the results of this study could provide the basis for a comprehensive profile of various ARGs among yak, beef, and dairy cattle in future.

## Introduction

Diverse and abundant antibiotics are used to control bacterial diseases and promote the growth of livestock, which may lead to antibiotic resistant bacteria (ARBs) that are widespread in the world [1]. Using of antibiotic as feed additives to promote growth in livestock farming has become a serious problem, and this is leading to increasing antibiotic resistance. Researchers have also shown that the antibiotic consumption in animals is already twice as much as used in humans [2]. According to the FDA, about 80% antimicrobials are used for animals in the USA [3]. In total, America used 14,600 tons of antimicrobials, and China used 97,000 tons of antimicrobials for animals in 2012 [3, 4]. In addition, the parent compounds and metabolites from antibiotics were detected in the excretion of animals because of incomplete metabolism and poor absorption in the gastrointestinal tract [5]. ARBs harboring ARGs can be delivered into the environment via animal feces. Feces directly or indirectly lead to the spread of ARGs in the environment, and there is a risk of eventual transmission to humans. Animal fecal bacteria communities are a vast reservoir of ARGs that can occur in commensals and pathogens of humans [6]. Studies on the diversity and abundance of drug-resistant genes in animal intestinal bacterial communities show that it will be very difficult for human to prevent and control animal bacterial diseases if the ARGs are transferred to and become prevalent in bacteria, which can become human pathogens. It is very important to study antibiotic resistance in gut microbiomes in animals for the effective prevention and control of bacterial diseases, the establishment of strategies to prevent the transfer of drug resistance in bacteria and the guidance of clinical drug use. At the same time, it is a great of significance for the public health and food safety.

China’s animal husbandry industry has developed rapidly, and it was the world’s largest consumer of antibiotics for animals in the year of 2010 [7]. Crowded enclosures and intensive production are the most profitable way for farming; however, with the growth of feeding density, infectious diseases can quickly spread among animals [8]. Therefore, controlling of bovine bacterial disease and researches of antibiotic drug resistance is very important. To reduce the risk of the spread of ARGs, researchers have devoted considerable effort to studying antibiotic resistance in herbivorous animals. Some researchers have even claimed the different level of antibiotics resistance can also be found in the different animal cohorts [9].

Studies have shown that ARGs can be transferred horizontally in the intestines of humans and animals, as well as in soil, sediment, and water [10–13]. The transfer rate of horizontal gene transfer (HGT) in the intestinal tract is 25 times higher than that it is in the environment [14]. Multiple mobile genetic elements (MGEs) carrying ARGs have been isolated from clinical ARBs. Through HTG, MGEs are important elements that drive the ARG dissemination. The frequency of transmission of HGT is much higher than other genes [15, 16]; however, the clear mechanisms of ARGs transmission in agriculture bovine are not understood.

A growing number of researchers focused on detecting the microbial diversity using the metagenomic approaches in the gastrointestinal tracts of various animals [17]. Rare and wild taxa in the microbiota of animal feces can be explored through metagenomic approach [16]. The purpose of this study is to achieve a comprehensive profile of 40 bovine fecal samples harboring ARGs and MGEs using a metagenomic approach to evaluate the difference and diversity of ARGs and MGEs among different species (yak, beef, and dairy cattle) raised under different conditions.

## Material and methods

### Sampling

A total of 40 fecal samples were collected from different breeding patterns and areas in China, including Xinjiang, Gansu, Qinghai, and Sichuan Provinces. Animals were maintained on the same diet for 28 days prior to the experiment to decrease variation. We randomly selected healthy bovine species in four provinces, and fresh fecal samples were collected and transported in liquid nitrogen. The fecal samples were placed at −80 °C until DNA extraction. The summary of the 40 fecal samples is shown in Table S1.

### DNA extraction and metagenome sequencing

DNA was extracted from fecal samples using the method of hexadecyltrimethylammonium bromide approach following the instructions provided [18]. DNA degradation degree and potential contamination were monitored on 1% agarose gels. DNA concentration was measured using Qubit® dsDNA Assay Kit in Qubit® 2.0 Fluorometer (Life Technologies, CA, USA) and the purity was measured using NanoPhotometer® spectrophotometer (IMPLEN, CA, USA). A total amount of 1 μg DNA per sample was used for shotgun library construction. Metagenomic sequencing was performed using Illumina Hiseq X ten platform with the sequencing strategy of Index 150 PE (paired-end sequencing). The specific processing steps were as follows: (1) reads which contained low-quality bases were removed; (2) reads in which the N base had reached a certain percentage were removed; (3) reads which shared the overlap above a certain portion with Adapter were removed. Over 12 Gb clean reads were detected via metagenomic approach in each sample, and the proportion of high-quality reads among all raw reads from each sample was no less 95%.

### Metagenome assembly and ORF prediction

After treatment, the high-quality clean data were assembled using SOAPdenovo2 assembly software [19]. Clean data from all samples were compared to Scaftigs using Soap Aligner [20]. Based on the Scaftigs of single samples and mixed assembly, gene catalogue was constructed to predict gene [20–23].

The open reading frame (ORF) within Scaftigs (≥500 bp) was MetaGeneMark. All of the ORF were filtered using CD-HIT with a minimum similarity of identity 95%, coverage 90% and were selected by the longest gene sequence as the standard for achieving nonredundant Unigenes [24, 25].

### Relative abundance analysis

Based on the number of mapped reads and the length of gene, the abundance information of each gene in each sample was calculated [26]. For each gene, “r” referred to the number of read pairs; “L” referred to the length of the corresponding gene; “G” referred to the relative abundance. The relative abundance of gene was calculated by the following formula:

$$G_k \,=\, \frac{{r_k}}{{L_k}} \cdot \frac{1}{{\mathop {\sum}\nolimits_{i \,=\, 1}^n {\frac{{r_i}}{{L_i}}} }}$$

### Taxonomy prediction

Unigenes sequences from each sample were then compared against NR database of NCBI (Version: 2018-01-02) by using DIAMOND with the parameters (blastp, *e* value ≤ 1e−5) [27]. Subsequently, we adopted the LCA algorithm (LCA parameters: mini-score 35, top percentage 10%) which was applied in the systematic classification of MEGAN software to make sure the species annotation information of sequence [28].

### Identification of ARGs

Nonredundant gene sets were compared with CARD database (https://card.mcmaster.ca/) using Resistance Gene Identifier software to annotate antibiotic resistant genes (blastp, *e* value ≤ 1e−30) [29–31]. The results of gene annotation were used to analyze the species corresponding to the ARG.

## Results

### Identification and taxonomy of ARGs

The metagenomic library was constructed for evaluating ARGs reservoir in the gut of yak, beef, and dairy cattle. A total of 40 fecal samples were tested using the Illumina platform and obtained 548.3662 Gbp of high quality and average on each sample was 13.0564 Gbp. And the detailed data summary was exhibited in Table S2.

**Fig. 1:**
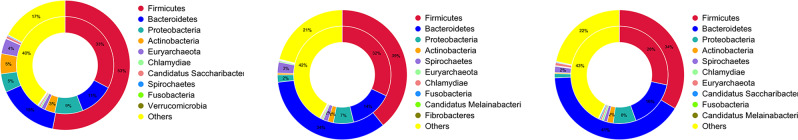
Comparison of the distribution of the ARGs and the bacterial gene sets at the phylum level of yak, beef, and dairy cattle, respectively. The inner circle is the species distribution of ARG, while the outer circle is the species distribution of all sample genes in the group

According to the annotation results compared with CARD, the species information corresponding to the drug-resistant genes were analyzed and the dominant flora carrying the drug-resistant genes are presented. Comparisons of the distribution of the bacterial gene sets and ARGs of yak at phylum level mainly showed that 53% vs. 33%, 15% vs. 11%, 5% vs. 9% to *Firmicutes*, *Bacteroidetes*, and *Proteobacteria*, respectively. For beef cattle, it is 39% vs. 32%, 34% vs. 15%, 2% vs. 6% to *Firmicutes, Bacteroidetes, and Proteobacteria*, respectively. For dairy cattle, it is 34% vs. 28%, 41% vs. 16% to *Firmicutes*, *Bacteroidetes*, respectively (Fig. 1). These asymmetric relationships suggest that *Firmicutes* are more likely to carry resistant genes in bovine fecal samples.

### Diversity and distribution of ARGs in gut

Approximately 85–99% of microbes cannot be cultivated in the laboratory, which limits our understanding of microbes including those with ARGs [32]; therefore, the metagenomic approach was used to investigate the distribution and diversity of drug resistance genes in the intestinal tract of bovine species. A total of 5,701,582 predictive genes were annotated after the original redundancy, and 1688 genes could be annotated by CARD database including 734 ARG types. To eliminate the differences in the numbers of ARGs caused by the differences in sample data, the numbers of ARGs annotated to each Gb of data in different groups were calculated. To be specific, the numbers of ARG/Gb in yak group (17.83 ± 2.67) were much lower than in groups of beef (18.28 ± 2.56) and dairy cattle (19.25 ± 1.77) (*P* < 0.001). The detailed information of ARGs in each sample is shown in Table S3a, b, c.

**Fig. 2:**
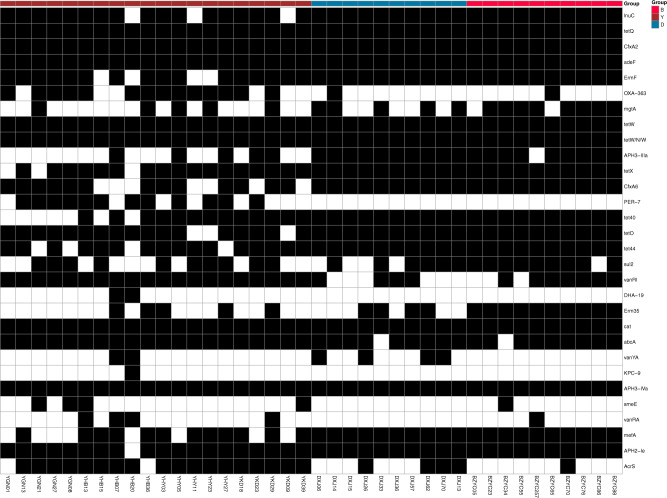
The heatmap of top 30 ARG distribution, the horizontal axis is the sample name, and the right vertical axis is the ARG name of resistance gene type. Black represents the ARO in the sample, while white represents the ARG in the sample

According to the abundance information of ARG in each sample, the top 30 ARGs are used to draw a heatmap (Fig. 2). The heatmap hierarchical clustering based on the relative abundance of each ARGs showed that yak samples were clustered individually, but they were not clustered with beef and dairy cattle samples.

### The relative abundance of ARGs in gut

**Fig. 3:**
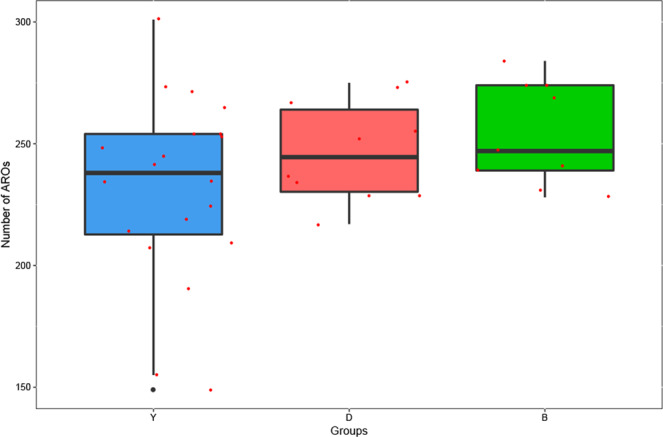
The difference on the number of ARGs among yak, beef, and dairy cattle is shown in the boxpot

There was some difference in ARGs number in the gut of yak, beef, and dairy cattle. Specially, the numbers of ARGs in yak group were lower than in other groups (Fig. 3). In addition, the relative abundance of ARGs was significantly higher in dairy cattle and beef than it was in yak (*P* < 0.001). The detailed relative abundance of ARGs in each sample is shown in Table S4.

**Fig. 4:**
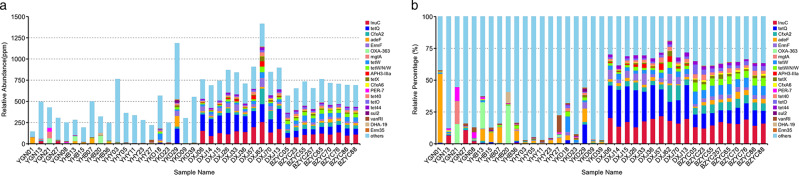
**a** Relative abundance of ARGs in each sample, and the unit PPM is the result of amplifying the original relative abundance data by 106 times; **b** Relative abundance of top 20 ARGs in all ARGs, and others represent the total relative abundance of non-top 20 ARGs

Starting from the relative abundance table of resistance genes, the ARGs content and percentage in each sample was calculated, and the ARGs results of the maximum abundance ranking top 20 were screened as diagram in Fig. 4a, b. It has seen that there was a difference in the relative abundance of ARGs among yak group and other groups. Specifically, the relative between abundance and percentage of the yak ARGs were different from they were in beef and dairy cattle.

**Fig. 5:**
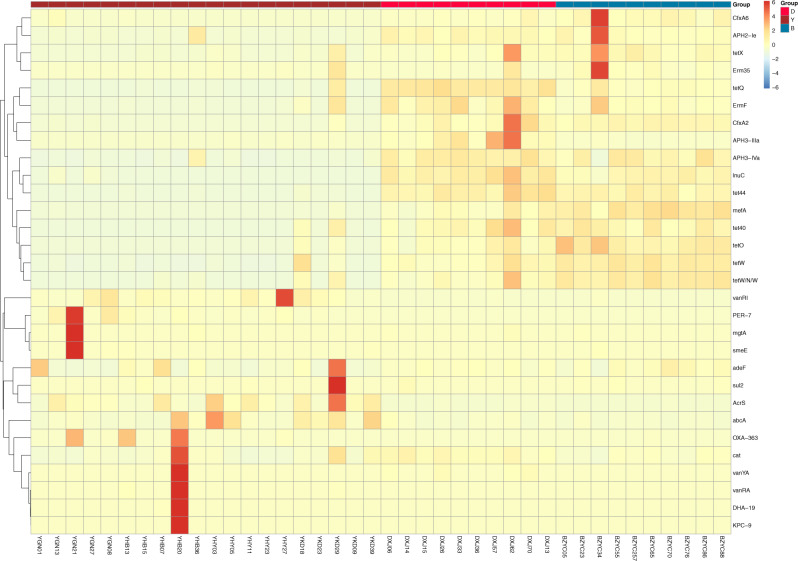
The top 30 ARG abundance clustering heatmap. The right vertical axis is the ARG name, the left vertical axis is the ARG clustering tree, and the corresponding value of the intermediate heatmap is the *Z* value of ARG relative abundance in each row after standardized processing

**Fig. 6:**
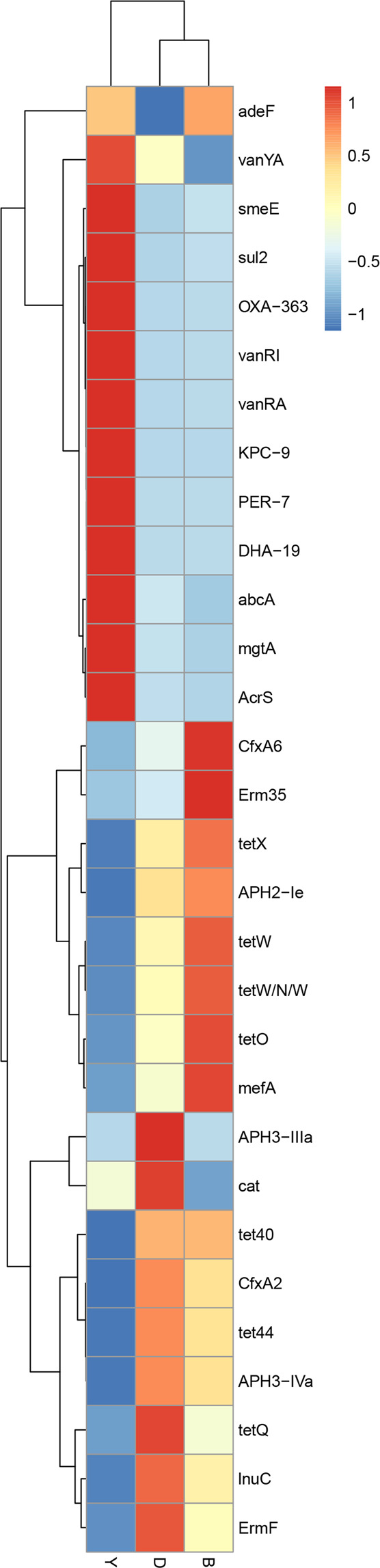
Heatmap variations of the relative abundance level of each top 30 ARGs subtype among the group of yak, beef, and dairy cattle. The right vertical axis is the name of ARG subtype, and the bottom of horizontal axis is the group name. The left vertical axis is cluster tree

The results showed that yak samples were clustered individually and were not clustered with beef and dairy cattle samples in the Figs. 5 and 6. The relative abundance of ARGs (tetX, tetQ, tet44, tet40, tetO, tetW, tetW/N/W) were higher in beef and dairy cattle than in yak. In addition, the relative abundance of ARGs (VanRI, VanYA, VanRA, DHA-19, OXA-363, PER-7, abcA, AcrS) was higher in yak than in beef and dairy cattle.

### Shared ARGs among yak faces, beef faces, and dairy cattle faces

**Fig. 7:**
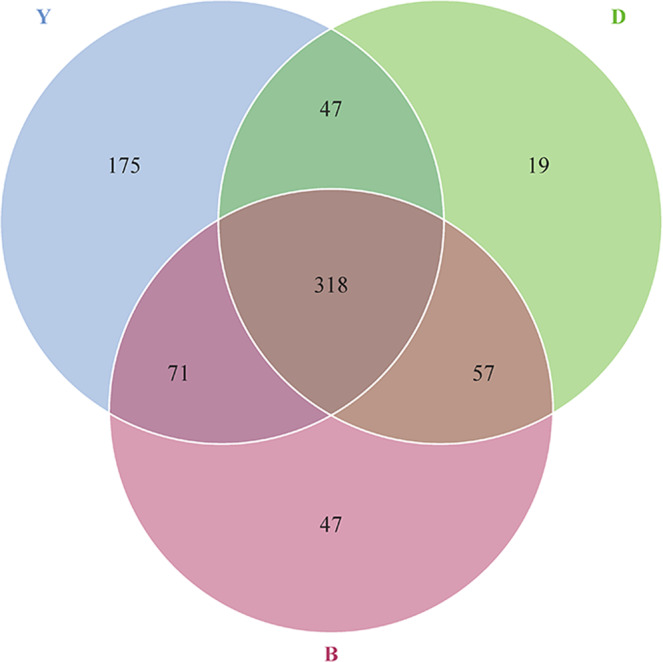
The Venn diagram showing the number of shared ARGs in yak gut, beef gut, and dairy cattle gut

**Fig. 8:**
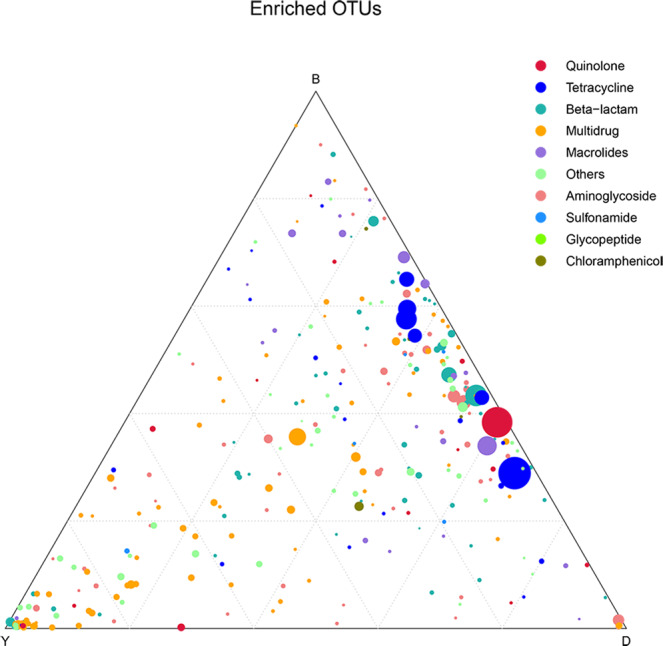
Ternary plot showing the abundance comparison of 318 shared ARGs in yak gut, beef gut, and dairy cattle gut. The sum of the abundance for one species ARG in these three types of gut was set as 100%. The percentage of each certain ARG in each gut is equal to its corresponding abundance which is divided by the abundance sum of this ARG in the three groups of gut

To detect the distribution of shared ARGs among yak, beef, and dairy cattle, the Venn diagram and Ternary plot were constructed. A sum of 318 ARGs was shared by faeces from yak, beef, and dairy cattle (Fig. 7). The differences in the abundance of 318 ARGs for different drugs in three groups were also analyzed in ternary plot (Fig. 8). The percentage of ARGs in each group of gut is equal to its corresponding abundance which are divided by the sum abundances of ARGs in three groups of guts. Among these data of shared ARGs, the abundance of tetracycline genes, quinolone genes, β-lactam genes, and macrolide genes were much higher in beef and dairy cattle than those in yak.

**Fig. 9:**
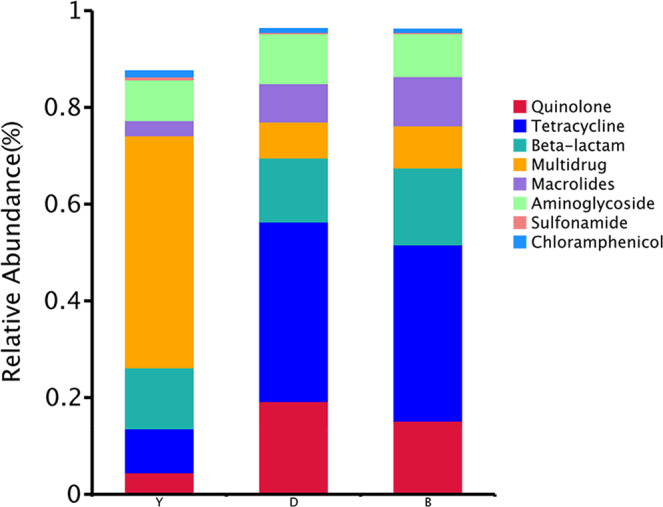
The relative abundance of shared ARGs types assigned to each major antibiotic class among the group of yak, beef, and dairy cattle

Moreover, the results demonstrated that two antibiotic classes of resistance genes consisting tetracyclines and β-lactams accounted for >50% of the total ARGs in beef and dairy cattle (Fig. 9), and the multidrug resistance genes accounted for nearly 50% in yak (Fig. 9).

### Occurrence and abundance of MGEs

**Fig. 10:**
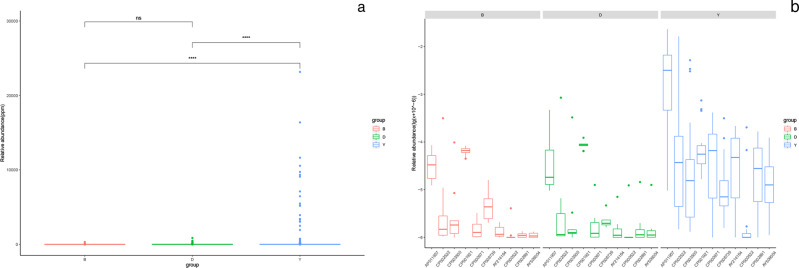
**a** The difference of relative abundance of mobile genetic elements (integron) among yak, beef, and dairy cattle is shown in the sigbox. (**P < 0.005, ***P < 0.001, ****P < 0.0001). **b** The difference of the abundance of top ten mobile genetic elements (integron) in the yak, beef, and dairy cattle

By comparing with the IS finder database, a total of 153,981 MGEs were annotated, and the differences among three groups were noted. Observational studies of mobile transfer elements illustrated that the abundance of integron in yak group was much higher than in beef and dairy cattle (*P* < 0.0001) (Fig. 10). There was no obvious difference between beef cattle and dairy cattle (*P* > 0.05). Moreover, the top ten most abundant integrons varied from yak to beef and dairy cattle, the AP011957 was the most abundant type in three groups, respectively (Fig. 10b).

## Discussion

The main aim of this study was to analyze the fecal resistome and bacterial community and to explore the effects of antibiotic selective pressure in yak, beef, and dairy cattle by using comparative metagenomic approaches. Notably, fecal samples from bovine species were collected by the same protocols and sequence platforms. The differences might exist because of different host animal species and batch effects might exist because of samples from multi-sites. In this study, we presumed that differential selective pressure of antibiotics might contribute to the difference in drug resistance in gut microbiota of yak, beef, and dairy cattle.

With the rapid decline in production costs and the increased use of antibiotics, more and more sub-therapeutic doses of antibiotics are used in the breeding industry to promote growth and prevent diseases [33, 34]. This usage partly explains why there are plenty ARGs detected in food animals. High population density produces high demands for animal production, which leads to the production of high-density feeding patterns. This is a close relationship in the use levels of antibiotics in animals to the population level [35]. The high-density feeding patterns potentially affect the increase in selective pressure of bacteria, thereby becoming resistant [7]. Generally, yak, beef, and dairy cattle are raised in different feeding patterns. Places at high altitudes of western China are home to yaks, where 90% of wild yak in the world are raised [36]. The yaks in the study are not completely wild, but cared by clinical veterinarians. They obtained food freely in a grazing environment. Whether the antibiotics were overused or improper used in yak cannot be fully known in this study. This may result in a relatively high abundance of an ARG in individual yak, thereby affecting the abundance of an ARG in the yak population. Generally, comparing with beef and dairy cattle, the yak is seldom exposed to antibiotics. In other words, yaks live in the low-density feeding patterns while beef and dairy cattle live in high-density feeding patterns. Animals under high-density feeding pattern have a higher risk of infection caused by antibiotics than those under low-density feeding pattern. High-density feeding pattern has even lead to zoonotic transmission, and it also acts as a huge reservoir of ARGs [37–39]. Compared with yak, beef, and dairy cattle exposed to more antibiotics are more likely to develop resistance. Therefore, the differences of abundance and diversity of ARGs among three groups may be associated with antimicrobial selective pressure caused by different density feeding patterns. The data of this study show compared with other two groups, the predominant ARGs types (top100) of yak is significant, and the abundance and diversity of ARGs in yak is lower than that in other groups, suggesting that the ARGs in the gut of yak, beef, and dairy cattle are affected by selective pressure. Researchers have also pointed out that the selective pressure generated by the excessive use of antibiotics in agricultural production can result in the emergence of drug resistance [40, 41], which leads to the persistence of resistance genes in the intestine [42]. In consequence, the problem of antibiotic resistance brought by antibiotic selective pressure due to high-density feeding pattern should not be ignored.

Further, the predominant shared ARGs types are tetracycline, quinolone, and β-lactam resistance genes in group of beef and dairy cattle via metagenomic approaches. These resistance genes are found in other species of animals, humans, and soils [16, 43–47]. Tetracycline and most quinolone resistance genes in beef and dairy cattle are obviously higher than those in yak group. The high percentage of these ARGs may not only be caused by antibiotic selective pressure, but also by horizontal transmission due to high-density feeding patterns. More and more evidence show that the MGEs are important in the mechanism of resistance to tetracycline and quinolones [48–51]. There is a correlation between these ARGs that might lead to multidrug resistance. For example, quinolone resistance genes existing in multidrug resistance plasmids are linked with other ARGs, like β-lactamase genes [52].

Recently, many studies have showed that MGEs carrying genes of varying activity may lead to selective drug resistance, indicating that MGEs are deemed as an important element in the prevalence of ARGs [53, 54]. Yaks are seldom exposed to antibiotics but still harbor resistant genes in their intestinal tract, which may be caused by MGEs for delivering ARGs. MGEs consist of insertion sequence, integron, transposons, plasmids, genomic islands, and so on. As an important element of MGEs involved in the development of resistance, integron can capture and integrate exogenous genes and spread ARGs horizontally in bacteria through transposons or plasmids via site-specific recombination [53, 55–57]. In our study, it is notable that the abundance of integron in yak gut is higher than that in other two groups, suggesting that high abundance of MGEs in yak may have much stronger ability to transfer ARGs and the potential to spread ARGs than that in beef and dairy cattle. In some cases, birds can carry and spread ARGs, or companion animals can spread ARGs across species through close contact [58]. Secondly, more and more reports have stated that soil, rivers and sediments are also huge reservoirs of ARGs, and animals in such an environment are easily exposed to ARGs. It partly explains why the intestinal microbiome of yaks carry ARGs even though they are not fed or treated with antibiotics. It is reported that integron-mediated ARGs can be transferred from one strain to another that derived from the bovine feces and storm water [59]. Importantly, more than 80 gene cassettes of class one integrons can become resistant to all β-lactam and all aminoglycosides [60]. Thus, capture systems of integrons allow bacteria to adapt to the challenges of antibiotic treatment regime. This integron-mediated interspecific transfer of ARGs poses a huge threat to antibiotic therapy in clinical use, so the importance of this capture system is not just a theoretical concern. This integron-mediated interspecific transfer of ARGs poses a huge threat to antibiotics in clinical use, so the importance of this capture system is not just a theoretical concern [61].

In conclusion, in this study, the data show that the difference among groups of yak, beef, and dairy cattle relating to predominant ARGs types is striking, and the abundance and diversity of ARGs in yak is lower than those in beef and dairy cattle. It implies that yaks exposed to fewer antibiotics may be less likely to develop resistance than beef, dairy cattle, and cows, because antibiotic selective pressure may due to different feeding patterns. However, two points are worth noting: Firstly, yaks are rarely fed or treated with antibiotics but still harbor a certain amount of ARGs in their intestine; secondly, high levels of integron are found in the intestinal tract of yaks. These conditions suggest that ARGs may be transmitted horizontally from the environment across species via integron-mediated transmission.

## Supplementary information

supplementary material

## Data Availability

The data used to support the findings of this study are included within the article and supplementary information files.
