# Correction: The occurrence of antibiotic resistance genes in the microbiota of yak, beef and dairy cattle characterized by a metagenomic approach

**DOI:** 10.1038/s41429-022-00590-y

**Published:** 2022-12-23

**Authors:** Weiwei Wang, Xiaojuan Wei, Lingyu Wu, Xiaofei Shang, Fusheng Cheng, Bing Li, Xuzheng Zhou, Jiyu Zhang

**Affiliations:** 1 Key Laboratory of New Animal Drug Project of Gansu Province, Lanzhou, Gansu Province 730050 PR China; 2 Key Laboratory of Veterinary Pharmaceutical Development, Ministry of Agriculture, Lanzhou, Gansu Province 730050 PR China; 3 Lanzhou Institute of Husbandry and Pharmaceutical Sciences, Chinese Academy of Agricultural Sciences, Lanzhou, Gansu Province 730050 PR China

**Keywords:** Antimicrobial resistance, Computational biology and bioinformatics

Correction to: *The Journal of Antibiotics*

10.1038/s41429-021-00425-2

published online 09 June 2021

In this article, the statement in the Funding information section was incorrectly given as ‘grant number 3187520’ and should have read ‘grant number 31872520’.

The original article has been corrected.

