# Supplementary material for: The occurrence of antibiotic resistance genes in the microbiota of yak, beef and dairy cattle characterized by a metagenomic approach
Source: J Antibiot (Tokyo). 2021 Jun 9;74(8):508–18. doi: 10.1038/s41429-021-00425-2 (PMC8313426; doi:10.1038/s41429-021-00425-2)
Supplement: Supplementary file 1 — supplementary material [file 41429_2021_425_MOESM1_ESM.docx]

Table S1 Basic information summary of 40 fecal samples

| Sample.ID | Sample sites | Sampling time | Sequencing technology | Data source |
| --- | --- | --- | --- | --- |
| YGN01 | Gannan, Gansu Province, China | August, 2018 | illumina Hiseq X ten  PE150 | This study |
| YGN08 |  |  |  |  |
| YGN13 |  |  |  |  |
| YGN21 |  |  |  |  |
| YGN27 |  |  |  |  |
| YHB07 | Haibei, Qinghai Province, China | August, 2018 |  |  |
| YHB13 |  |  |  |  |
| YHB15 |  |  |  |  |
| YHB20 |  |  |  |  |
| YHB36 |  |  |  |  |
| YHY03 | Hongyuan, Sichuan Province, China | August, 2018 |  |  |
| YHY05 |  |  |  |  |
| YHY11 |  |  |  |  |
| YHY23 |  |  |  |  |
| YHY27 |  |  |  |  |
| YKD09 | Kangding, Sichuan Province, China | August, 2018 |  |  |
| YKD18 |  |  |  |  |
| YKD23 |  |  |  |  |
| YKD29 |  |  |  |  |
| YKD39 |  |  |  |  |
| BZYC05 | Zhangye, Gansu Province, China | December, 2018 |  |  |
| BZYC23 |  |  |  |  |
| BZYC34 |  |  |  |  |
| BZYC55 |  |  |  |  |
| BZYC65 |  |  |  |  |
| BZYC70 |  |  |  |  |
| BZYC76 |  |  |  |  |
| BZYC86 |  |  |  |  |
| BZYC88 |  |  |  |  |
| BZYC257 |  |  |  |  |
| DXJ06 | Yili, Xinjiang Province, China | September, 2018 |  |  |
| DXJ13 |  |  |  |  |
| DXJ14 |  |  |  |  |
| DXJ15 |  |  |  |  |
| DXJ26 |  |  |  |  |
| DXJ33 |  |  |  |  |
| DXJ36 |  |  |  |  |
| DXJ57 |  |  |  |  |
| DXJ62 |  |  |  |  |
| DXJ70 |  |  |  |  |

Table S2 Metagenome sequencing data summary for 40 fecal samples

| Sample | ORF. No | Raw Data | Clean Data | Clean_Q20 | Clean_Q30 | Effective (%) | Length(Mbp) |
| --- | --- | --- | --- | --- | --- | --- | --- |
| YGN01 | 103,107 | 13,296.62 | 13,235.14 | 96.60 | 90.98 | 99.538 | 62.89 |
| YGN08 | 228,481 | 12,761.62 | 12,710.47 | 96.32 | 90.22 | 99.599 | 138.64 |
| YGN13 | 338,772 | 13,296.62 | 13,235.14 | 96.60 | 90.98 | 99.538 | 193.7 |
| YGN21 | 214,647 | 12,905.03 | 12,884.94 | 95.66 | 89.08 | 99.844 | 124.19 |
| YGN27 | 337,739 | 12,918.12 | 12,874.48 | 96.13 | 89.85 | 99.662 | 197.28 |
| YHB07 | 211,614 | 13,165.90 | 13,076.37 | 96.01 | 89.69 | 99.320 | 126.48 |
| YHB13 | 318,948 | 14,181.38 | 14,053.63 | 95.95 | 89.63 | 99.099 | 185.3 |
| YHB15 | 271,456 | 12,448.38 | 12,417.76 | 96.53 | 90.66 | 99.754 | 159.18 |
| YHB20 | 125,936 | 12,849.35 | 12,828.86 | 95.38 | 88.41 | 99.841 | 77.95 |
| YHB36 | 261,238 | 12,729.96 | 12,678.13 | 95.69 | 89.11 | 99.593 | 168.4 |
| YHY03 | 306,830 | 12,818.65 | 12,792.12 | 95.74 | 89.21 | 99.793 | 179.52 |
| YHY05 | 309,149 | 13,727.43 | 13,701.41 | 95.62 | 89.07 | 99.810 | 173.19 |
| YHY11 | 292,499 | 12,688.79 | 12,644.75 | 96.47 | 90.47 | 99.653 | 168.33 |
| YHY23 | 333,542 | 12,807.76 | 12,775.95 | 96.27 | 90.16 | 99.752 | 195.5 |
| YHY27 | 102,528 | 11,532.03 | 11,510.52 | 95.25 | 88.27 | 99.813 | 69.91 |
| YKD09 | 387,314 | 13,284.20 | 13,247.24 | 96.69 | 91.11 | 99.722 | 220.2 |
| YKD18 | 400,242 | 12,830.50 | 12,804.64 | 95.25 | 88.30 | 99.798 | 261.66 |
| YKD23 | 350,882 | 12,587.21 | 12,556.92 | 96.19 | 90.12 | 99.759 | 204.58 |
| YKD29 | 308,355 | 14,068.96 | 14,024.55 | 95.70 | 89.11 | 99.684 | 216.44 |
| YKD39 | 410,443 | 13,183.05 | 13,160.56 | 95.73 | 89.28 | 99.829 | 248.03 |
| BZYC05 | 441,805 | 14,454.87 | 14,434.56 | 95.26 | 88.31 | 99.859 | 258.73 |
| BZYC23 | 422,649 | 13,381.59 | 13,362.42 | 96.03 | 89.79 | 99.857 | 252.42 |
| BZYC34 | 414,255 | 14,614.76 | 14,569.89 | 95.85 | 89.37 | 99.693 | 256.59 |
| BZYC55 | 362,786 | 14,434.61 | 14,403.68 | 95.98 | 89.67 | 99.786 | 213.72 |
| BZYC65 | 327,315 | 13,087.38 | 13,061.29 | 95.68 | 89.11 | 99.801 | 197.14 |
| BZYC70 | 343,762 | 13,057.71 | 13,025.64 | 95.81 | 89.33 | 99.754 | 199.21 |
| BZYC76 | 397,160 | 11,713.15 | 11,686.93 | 96.05 | 89.79 | 99.776 | 250.37 |
| BZYC86 | 164,545 | 12,810.33 | 12,780.99 | 96.20 | 90.09 | 99.771 | 106.43 |
| BZYC88 | 359,977 | 13,424.57 | 13,403.38 | 96.25 | 90.19 | 99.842 | 207.37 |
| BZYC257 | 360,875 | 13,370.17 | 13,338.22 | 96.14 | 89.98 | 99.761 | 207.53 |
| DXJ06 | 112,982 | 12,472.89 | 12,443.58 | 96.19 | 90.07 | 99.765 | 59.44 |
| DXJ13 | 188,190 | 12,039.84 | 12,005.33 | 95.71 | 89.12 | 99.713 | 108.51 |
| DXJ14 | 183,505 | 12,973.12 | 12,953.99 | 95.88 | 89.38 | 99.853 | 102.03 |
| DXJ15 | 58,448 | 12,332.21 | 12,306.68 | 95.88 | 89.50 | 99.793 | 32.97 |
| DXJ26 | 290,414 | 12,766.71 | 12,741.52 | 95.91 | 89.53 | 99.803 | 156.83 |
| DXJ33 | 150,650 | 14,164.49 | 14,145.71 | 95.88 | 89.46 | 99.867 | 81.32 |
| DXJ36 | 125,050 | 12,678.36 | 12,652.59 | 95.66 | 89.03 | 99.797 | 66.02 |
| DXJ57 | 125,141 | 13,113.23 | 13,088.65 | 95.45 | 88.65 | 99.813 | 69.45 |
| DXJ62 | 161,917 | 12,930.14 | 12,906.95 | 95.22 | 88.04 | 99.821 | 91.89 |
| DXJ70 | 247,765 | 13,167.06 | 13,136.36 | 95.67 | 89.11 | 99.767 | 133.26 |

Table S3a Detailed information of drug resistance genes in each sample (yak)

| Samples | Clean reads | Data size(Gb) | Number of ARGs | Number of ARGs/Gb |
| --- | --- | --- | --- | --- |
| YGN01 | 40,752,434 | 12.23 | 149 | 12.18 |
| YGN08 | 42,368,234 | 12.71 | 234 | 18.41 |
| YGN13 | 44,117,134 | 13.24 | 271 | 20.47 |
| YGN21 | 42,949,800 | 12.88 | 190 | 14.75 |
| YGN27 | 42,914,934 | 12.87 | 253 | 19.66 |
| YHB07 | 43,587,900 | 13.07 | 219 | 16.76 |
| YHB13 | 46,845,434 | 14.05 | 273 | 19.43 |
| YHB15 | 41,392,534 | 12.42 | 207 | 16.67 |
| YHB20 | 42,762,867 | 12.83 | 155 | 12.08 |
| YHB36 | 42,260,434 | 12.68 | 214 | 16.88 |
| YHY03 | 42,640,400 | 12.79 | 254 | 19.86 |
| YHY05 | 45,671,367 | 13.70 | 301 | 21.97 |
| YHY11 | 42,149,167 | 12.64 | 248 | 19.62 |
| YHY23 | 42,586,500 | 12.78 | 241 | 18.86 |
| YHY27 | 44,104,034 | 13.23 | 235 | 17.76 |
| YKD09 | 44157467 | 13.25 | 254 | 19.17 |
| YKD18 | 42,682,134 | 12.80 | 224 | 17.5 |
| YKD23 | 41,856,400 | 12.56 | 245 | 19.51 |
| YKD29 | 46,748,500 | 14.02 | 209 | 14.91 |
| YKD39 | 43,868,534 | 13.16 | 265 | 20.14 |
| Mean±SD | 43320810.40±1620650.66 | 13.00±0.48 | 232.05±37.89 | 17.83±2.67 |

Table S3b Detailed information of drug resistance genes in each sample (beef)

| samples | Clean reads | Data size(Gb) | Number of ARGs | Number of ARGs/Gb |
| --- | --- | --- | --- | --- |
| BZYC05 | 48,115,200 | 14.43 | 239 | 16.56 |
| BZYC23 | 44,541,400 | 13.36 | 247 | 18.49 |
| BZYC34 | 48,566,300 | 14.57 | 180 | 12.35 |
| BZYC55 | 48,012,267 | 14.40 | 284 | 19.72 |
| BZYC65 | 43,537,634 | 13.06 | 274 | 20.98 |
| BZYC70 | 43,418,800 | 13.03 | 274 | 21.03 |
| BZYC76 | 43,538,534 | 13.06 | 231 | 17.69 |
| BZYC86 | 42,603,300 | 12.78 | 228 | 17.84 |
| BZYC88 | 44,677,934 | 13.40 | 269 | 20.07 |
| BZYC257 | 44,460,734 | 13.34 | 241 | 18.07 |
| Mean±SD | 45147210.30±2221201.38 | 13.54±0.67 | 246.7±30.76 | 18.28±2.56 |

Table S3c Detailed information of drug resistance genes in each sample (dairy cattle)

| samples | Clean reads | Data size(Gb) | Number of ARGs | Number of ARGs/Gb |
| --- | --- | --- | --- | --- |
| DXJ06 | 41,478,600 | 12.44 | 217 | 17.44 |
| DXJ13 | 40,017,736 | 12.04 | 273 | 22.67 |
| DXJ14 | 43,179,967 | 12.95 | 234 | 18.07 |
| DXJ15 | 41,022,267 | 12.31 | 229 | 18.60 |
| DXJ26 | 42,471,734 | 12.74 | 275 | 21.59 |
| DXJ33 | 47,152,367 | 14.15 | 255 | 18.02 |
| DXJ36 | 42,175,300 | 12.65 | 237 | 18.74 |
| DXJ57 | 43,628,834 | 13.09 | 229 | 17.49 |
| DXJ62 | 43,023,167 | 12.91 | 252 | 19.52 |
| DXJ70 | 43,787,867 | 13.14 | 267 | 20.32 |
| Mean±SD | 42793783.90±1942082.84 | 12.84±0.58 | 246.80±20.45 | 19.25±1.77 |

Table S4 Relative abundance of ARGs in individuals

| Sample | Yak | Sample | Yak | Sample | Beef | Sample | dairy cattle |
| --- | --- | --- | --- | --- | --- | --- | --- |
| YGN01 | 1.43E-4 | YHY03 | 7.62E-4 | BZYC05 | 5.66E-4 | DXJ06 | 7.58E-4 |
| YGN08 | 2.50E-4 | YHY05 | 3.62E-4 | BZYC23 | 6.51E-4 | DXJ13 | 8.95E-4 |
| YGN13 | 2.80E-4 | YHY11 | 3.33E-4 | BZYC34 | 8.52E-4 | DXJ14 | 6.88E-4 |
| YGN21 | 4.26E-4 | YHY23 | 2.77E-4 | BZYC55 | 6.54E-4 | DXJ15 | 7.42E-4 |
| YGN27 | 3.03E-4 | YHY27 | 2.17E-4 | BZYC65 | 7.60E-4 | DXJ26 | 8.69E-4 |
| YHB07 | 4.98E-4 | YKD09 | 3.01E-4 | BZYC70 | 7.14E-4 | DXJ33 | 8.40E-4 |
| YHB13 | 2.80E-4 | YKD18 | 5.66E-4 | BZYC76 | 7.04E-4 | DXJ36 | 7.08E-4 |
| YHB15 | 1.91E-4 | YKD23 | 2.48E-4 | BZYC86 | 6.92E-4 | DXJ57 | 9.06E-4 |
| YHB20 | 3.20E-4 | YKD29 | 1.19E-4 | BZYC88 | 6.89E-4 | DXJ62 | 1.41E-4 |
| YHB36 | 2.45E-4 | YKD39 | 5.51E-4 | BZYC257 | 6.54E-4 | DXJ70 | 8.45E-4 |
